# Cefazolin population pharmacokinetics in children undergoing maintenance hemodialysis for kidney failure

**DOI:** 10.1128/aac.00451-25

**Published:** 2025-10-02

**Authors:** Romain Berthaud, Saïk Urien, Saoussen Krid, Frantz Foissac, Mehdi Oualha, Michaël Thy, Olivia Boyer, Agathe Béranger, Déborah Hirt, Sihem Benaboud, Jean-Marc Tréluyer, Naïm Bouazza

**Affiliations:** 1 Université Paris Cité, INSERM, Pharmacologie et Evaluations des Thérapeutiques chez l'Enfant et la Femme Enceinte, Paris, France; 2 Centre d’Investigation Clinique 1419, Inserm, AP-HP, Hôpital Necker-Enfants Malades 246596, Paris, France; 3 Service de Néphrologie Pédiatrique, AP-HPH, Hôpital Necker-Enfants Malades, Paris, France; 4 Unité de Recherche Clinique Necker Cochin, AP-HP, Hôpital Tarnier, Paris, France; 5 Unité de Réanimation et Surveillance Continue Pédiatrique, AP-HP, Hôpital Necker-Enfants Malades, Paris, France; 6 Université Sorbonne Paris Nord, INSERM, UMR1137 IAME, Paris, France; 7 Unité de Réanimation, AP-HP, Hôpital Bichât Claude-Bernard, Paris, France; 8 Université Paris Cité, INSERM, U1163 Institut Imagine, Paris, France; 9 Département de Pharmacologie, AP-HP, Hôpital Cochin 26935, Paris, France; Providence Portland Medical Center, Portland, Oregon, USA

**Keywords:** cefazolin, population pharmacokinetics, children, renal replacement therapy, hemodialysis, maintenance, kidney failure, dialysis

## Abstract

We aimed to develop a population pharmacokinetic model of cefazolin in children undergoing maintenance hemodialysis for kidney failure and simulate dosing regimens to optimize patients’ exposure. Cefazolin plasma concentrations were quantified using high-performance liquid chromatography. A non-linear mixed-effect modeling approach was used to investigate cefazolin pharmacokinetics. Optimal dosing regimens were determined using Monte Carlo simulations targeting 100% of the time a free plasma concentration four times above the minimum inhibitory concentration (MIC) and total plasma concentration less than 80 mg/L (100% fT > 4 × MIC and *C* < 80 mg/L). Eighty-three samples were analyzed from six patients aged 1.3–14.6 years and weighing 11.4–51 kg. A one-compartment model with first-order elimination best fitted the data, with a significant between-subject variability (BSV). Body weight (BW), using the allometric rule, was a significant predictor of residual elimination clearance (CL) and volume of distribution (Vd), while dialysis membrane surface area (DMSA) explained almost all the BSV on dialysis clearance (CLdial). The parameter relationships were Vd_*i*_(L) = 14.6 × (BW*_i_*/70), CL_i_(L/h) = 0.186 × (BW*_i_*/70)^0.75^, CLdial_*i*_(L/h)=1.98 × (DMSA*_i_*/1)^1.26^, CLtot_i_ = CL*_i_ +* CLdial_DMSA_*_i_*, where the subscript *i* denotes the *i*^th^ patient’s covariate value. Dosing regimens were simulated for six CL ranges, targeting two concentration intervals: 40–80 mg/L and 20–80 mg/L for MIC of 2 and 1 mg/L, respectively. In children on maintenance hemodialysis for kidney failure, due to the persistence of a large unexplained BSV on CL that substantially affects them, optimal dosing regimen would not be accurately determined a priori and should be individually determined using therapeutic drug monitoring, taking BW, DMSA, and CL into account.

**CLINICAL TRIALS:**

This study is registered with ClinicalTrials.gov as NCT02539407.

## INTRODUCTION

Cefazolin is a narrow-spectrum first-generation cephalosporin active against methicillin-susceptible *Staphylococcus aureus* (MSSA). It is a time-dependent antibiotic administered intravenously, not metabolized, and eliminated in active form in the urine. It is highly bound (around 80%) to serum albumin (1).

Compared to the general population, infection-related mortality is much greater in adult patients who receive dialysis (2–4). *Staphylococcus aureus* (SA) is the leading pathogen causing severe infections in adults on maintenance hemodialysis, accounting for over 30% of bloodstream infections (5–9) and is associated with vascular access site infections (7, 8, 10). SA bacteremia-related morbidity and mortality tend to increase in adults undergoing maintenance hemodialysis, with MSSA accounting for more than 50% of isolated SA (7, 8). In non-dialysis children, SA is one of the leading invasive bacterial pathogens, and the rate of MSSA bloodstream infections is progressively rising (11, 12). The few data available in hemodialysis-dependent children emphasize the major role of SA in vascular access-related bloodstream infections, when the vascular access is a central venous catheter (12–14), which is the predominant vascular access choice in this population (13, 15).

Cefazolin is the appropriate antibiotic to treat MSSA bacteremia with a superior efficacy to that of vancomycin in non-dialysis (16–18) and in hemodialysis-dependent adult patients (19–21). When compared to other anti-staphylococcal penicillins, cefazolin was found to be at least equally as effective for the treatment of patients with SA bacteremia, while being associated with less nephrotoxicity (22). Guidelines for the management of intravascular catheter-related infection in adults recommend that patients who receive empirical vancomycin and who are found to have a catheter-related bloodstream infection due to MSSA should be switched to cefazolin (23). These guidelines are extrapolated to children due to the lack of specific data in this population.

As cefazolin pharmacokinetics (PK) has been scarcely studied in children, pediatric dosing regimens are extrapolated from adult studies with a risk of inadequate concentrations (24–26). Kidney failure is the terminal phase of chronic kidney disease (CKD) progression, with glomerular filtration rate (GFR) lower than 15 mL/min/1.73 m^2^ (0.9 L/h/1.73 m^2^). As a drug with renal elimination, cefazolin PK parameters are significantly altered by reduced GFR, particularly its clearance is reduced (27–30). Moreover, during hemodialysis sessions, a dialysis clearance, itself variable (27), is added to the patient residual elimination clearance (non-renal + residual renal clearance) of cefazolin.

This, added to organ maturation and growth effects, will result in large and unpredictable between- and within-subject variabilities, which may cause under- or over-dosing leading to inefficiency and bacterial resistance or neurological toxicity, respectively (31–36).

Cefazolin studies in patients with kidney failure undergoing maintenance hemodialysis are rare, and advances in hemodialysis techniques have made them obsolete (21, 37–41).

This study aimed to investigate individual characteristics that can influence cefazolin PK in children undergoing maintenance hemodialysis for kidney failure by building a population PK model to help individualize treatment using Bayesian forecasting.

## MATERIALS AND METHODS

### Patients and settings

This prospective study was conducted between January 2018 and December 2019, in the Pediatric Nephrology department of Necker-Enfants Malades university hospital in Paris, France, as part of the wider Optimome study.

All children (aged < 18 years old) undergoing maintenance hemodialysis for kidney failure and receiving intravenous cefazolin were included. Patients were excluded if parents or caregivers did not consent to their participation.

The following patients’ baseline characteristics were recorded: body weight (BW), height, age, sex, pathology or cause of kidney failure, reason for cefazolin prescription, type of infection, cefazolin dosing regimen, serum albumin concentration and maintenance hemodialysis characteristics including dialysis machines, type and size of dialysis membranes, blood flow rate, dose of dialysis, ultrafiltration volume, type of vascular access, and renal replacement therapy (RRT) techniques.

The fat-free mass (FFM) was calculated with the following formula (42):


$$FFM=WHS_{max}\times HT^{2}\times \left\{BW/\left[\left(WHS_{50}\times HT^{2}\right)+BW\right]\right\}$$

where WHS_max_ is the maximum FFM for a given height (HT, m) and WHS_50_ is the total BW (kg) value when FFM is half of WHS_max_. Values of WHS_max_ are 42.92 and 37.99 kg/m² and values of WHS_50_ are 30.93 and 35.98 kg/m² for males and females, respectively.

### Study design

According to the local protocol, cefazolin (1 g powder, Mylan) was diluted with sodium chloride 0.9% or glucose 5% solution (Fresenius Kabi) to obtain 20 mg/mL standard solutions. Cefazolin was administered by 30–60 min intravenous intermittent infusions every 6, 8, 12, 24, or 48 h using a programmable electronic syringe pump (Orchestra DPS, Fresenius Kabi). Blood samples were collected, as part of the patient’s routine clinical care, to adapt their cefazolin dosing regimens in order to optimize efficacy and to prevent toxicity. Dosing regimen and times of administrations were defined following local experience and could be modified during the treatment period at the discretion of the treating physician, in close collaboration with a pharmacologist, based on cefazolin plasma assay results.

The occurrence of adverse effects was monitored daily by the pharmacovigilance team.

### Maintenance hemodialysis

Maintenance hemodialysis was performed using three dialysis machines: Gambro AK200 Ultra S (Gambro, Sweden, Stockholm), Nikkiso DBB-05 (Nikkiso Co., Ltd., Tokyo, Japan) and Fresenius 5008S CorDiax (Fresenius Medical Care, Bad Homburg, Germany); under three different techniques: hemodialysis, pre-dilution hemofiltration, and post-dilution hemofiltration.

Maintenance hemodialysis modalities, including type and surface area of dialysis membranes, blood flow rate, ultrafiltration volume, type of vascular access, and RRT technique, were prescribed by the treating physician according to local procedures.

A single bolus of enoxaparin 0.5–1 mg/kg was administered at the beginning of each dialysis session to prevent clotting in the blood circuit.

### Cefazolin and albumin assays

Cefazolin total plasma concentrations were quantified in the pharmacology laboratory at Cochin Hospital (Paris, France) using a validated high-performance liquid chromatography with ultraviolet detection. Samples were centrifuged (4,000 *g*, 5 min) and 200 µL of plasma samples were mixed with 50 µL Doripenem (internal standard), then proteins were precipitated using 500 µL acetonitrile. After centrifugation (13,000 *g*, 5 min), the supernatant was evaporated to dryness with nitrogen at 30°C and reconstituted with 100 µL of 3-morpholinopropanesulfonic acid buffer (100 nM, pH 6.8 adjusted with 2.5 N sodium hydroxide).

Finally, 20 µL of each sample was injected into the chromatographic system. The separation was carried out on a Kinetex C18 (150 × 4.6 mm, 5 µm, Phenomenex). The mobile phase consisted of 95% ammonium acetate buffer (20 mM, pH 6.5) and 5% of methanol with a flow rate of 1 mL/min. The detection wavelength was set at 280 nm for cefazolin. The calibration range was 0.5–200 mg/L, with inter- and intra-assay precision and accuracy <15% (43).

The method was validated according to the European Medicines Agency guidelines for bioanalytical method validation (43).

Serum albumin concentration was determined by immunoturbidimetry (Abbott Alinity, Chicago, USA).

### Population PK modeling

Cefazolin concentrations were modeled using a non-linear mixed-effect modeling software (Monolix, version 2023R1, http://lixoft.com). Parameters were estimated by computing their maximum likelihood estimator using stochastic approximation expectation maximization algorithm. To define the basic structural model, both one- and two-compartment structural models with first-order elimination were tested.

The PK parameters were CL, Vd and CLdial, which stand for the residual elimination clearance, central volume of distribution, and dialysis clearance, respectively.

The between-subject variabilities (BSV, *ω*, square root of the between-subjects variance *ω*²) were ascribed to an exponential distribution. Additive, proportional, and combined models were tested to describe the residual variability (*σ*). Demographic and clinical characteristics that could affect cefazolin PK were investigated as covariates: BW, FFM, maintenance hemodialysis characteristics including type and size of dialysis membranes, blood flow rate, ultrafiltration volume, type of vascular access, and RRT techniques (hemodialysis, pre-dilution hemofiltration, and post-dilution hemofiltration).

Because the drug is around 80% albumin-bound in plasma, the effect of serum albumin concentration was investigated assuming that drug distribution and elimination depend on the unbound drug concentration. Alternative relationships were examined by linear and nonlinear protein binding regression, as previously described (44).

Categorical covariates were assessed as follows:


$$\theta_{i}=\theta_{pop}\times \theta^{cov}$$

where *θ_i_* is the individual PK parameter for the *i*^th^ patient, *θ*_pop_ is the median population value for parameter of the group for which the covariate is equal to 0, *θ* is the covariate parameter and *cov* is the category 0 or 1 for the covariate.

Continuous covariates were associated using a power function:


$$\theta_{i}=\theta_{pop}\;\times {\left\{{cov}_{i}/\left[med\left(cov\right)\right]\right\}}^{\beta }$$

where *cov_i_* is the covariate value for the *i*^th^ patient, med(*cov*) is the median value of the covariate and *β* is the exponent.

The effect of BW was assessed according to the allometric rule with *β* values fixed at 0.75 for CL and CLdial and 1 for Vd (45).

The Bayesian information criterion (BIC) was used to assess the models. PK parameters were properly estimated if the relative standard errors (RSE) were <50%. The effect of a covariate was retained if it caused a decrease in the BIC and reduced the corresponding BSV. The goodness-of-fit plots of each model were evaluated by visual inspection of observed-predicted (population and individual) concentration scatter plots and normalized prediction distribution error (NPDE) versus time/predicted concentration scatter plots. From the final model, 500 Monte Carlo simulations per patient were performed to draw the prediction-corrected visual predictive check (pc-VPC) and compute the NPDE metrics, whose mean, variance, and distribution must not be different from 0, 1, and a normal distribution, respectively, to evaluate the mode (46). Diagnostic graphics and other statistics were obtained using the R software.

### Cefazolin concentration target and dosing regimen simulation

The accepted targets for cefazolin for critically ill patients are, considering a free cefazolin fraction of 20%, (i) trough free plasma concentration (intermittent administration) (fCmin) or free steady-state plasma concentration (continuous administration) (fCss) ≥ 4 × minimum inhibitory concentration (MIC), (ii) trough total plasma concentration (intermittent administration) (Cmin) or total steady-state plasma concentration (continuous administration) (Css) < 80 mg/L for documented infections, and (iii) Cmin 40–80 mg/L and Css 40–80 mg/L for non-documented infections (36). fCmin and fCss ≥4 × MIC equates to Cmin and Css ≥ 20 × MIC.

MICs were not available for the pathogens identified in our study. Two MIC thresholds were used for dosing regimen simulations: 2 mg/L, which is the EUCAST (European Committee on Antimicrobial Susceptibility Testing) epidemiological cut-off values (ECOFFs) for *Staphylococcus aureus*; and 1 mg/L which seemed relevant as it represents 95% of the wide strains of MSSA (MIC ≤ 2 mg/L ; EUCAST). Only 5% of the wide strains of MSSA have an MIC > 1 mg/L and ≤2 mg/L. The two targeted ranges were then Cmin or Css 40–80 mg/L (MIC 2 mg/L) and 20–80 mg/L (MIC 1 mg/L).

From the final model, 500 Monte Carlo simulations were performed using the R software to determine the probability of target attainment including both structural parameters, BSV, and the residual variability. The objective was 100% of the time within the target ranges. Multiple intermittent dosing regimens were simulated with several doses and intervals between doses.

## RESULTS

### Patients

Eighty-five samples from six patients were available. Two samples were discarded as outliers. There were no concentrations below the limit of quantification (BLQ). Patients’ baseline characteristics are summarized in Table 1.

**TABLE 1:** Patients' characteristics^*a*^^,^^*b*^

	Patient 1	Patient 2	Patient 3	Patient 4	Patient 5	Patient 6
Age, years	11.0	11.2	14.6	1.3	4.5	14.3
Gender	Male	Male	Male	Male	Male	Female
Body weight, kg	23.2	24.5	47.5	11.4	12.3	51.0
Height, cm	135	136	170	83	89	164
Number of dialysis sessions, *n*	1	10	3	13	6	13
Duration of patient involvement, days	4	17	7	15	10	31
Cause of kidney failure	Hemolytic uremic syndrome	Hemolytic uremic syndrome	Unknown	Denys-Drash syndrome	Schimke Immuno-osseous Dysplasia	Unknown
Reason for cefazolin prescription	MSSA infection	MSSA infection	MSSA infection	MSSA infection	MSSA infection	MSSA infection
Type of infection	Bloodstream infection	Bloodstream infection	Bloodstream infection	Bloodstream infection	Bloodstream infection	Bloodstream infection
Dialysis membrane surface area, m^2^	1	1	1.5	0.2	0.3	1.4/1.7
Mean blood flow rate, mL/min	130	195	188	117	91	260
Mean ultrafiltration volume, mL	370	579	1400	399	303	1715
RRT technique	Hemodialysis	Hemodialysis	Hemodialysis	Hemodialysis	Hemodialysis	Hemodialysis/post-dilution hemofiltration
Type of vascular access	CVC	CVC/AV fistula (bipuncture)	AV fistula (uni/bipuncture)	CVC	CVC	AV fistula (uni/bipuncture)
Mean serum albumin concentration, g/L (min–max)	26.1 (N/A)	29.2 (28–29.9)	47 (46.5–48.1)	37.2 (34.1–38.8)	26.3 (23.2–29.1)	34.6 (31.5–39.6)
Mean fat-free mass, kg	22.8	23.8	43.0	10.3	11.4	35.1
Mean cefazolin dose per infusion, mg/kg (min–max)	25.9 (N/A)	19.7 (16.3–24.5)	15.4 (10.5–25.3)	11.4 (7.0–26.3)	8 (5–10)	12.6 (4.9–19.8)

^*a*^ AV, arteriovenous; CVC, central venous catheter; N/A, not applicable; RRT, renal replacement therapy.

^*b*^ Duration of patient involvement, from the first cefazolin infusion to the last cefazolin concentration sample.

All patients were undergoing maintenance hemodialysis for more than 6 months at the time of inclusion. The median [Q1–Q3] (min–max) number of dialysis sessions was 8 [4–12] (1–13). Dialysis membranes used were Sureflux 30L, Elisio 15H, and Elisio 17H (Nipro Medical Europe, Mechelen, Belgium), and FXpaed, Fx50, and Fx60 (Fresenius Medical Care, Bad Homburg, Germany). Three types of vascular access were used: arteriovenous fistula using unipuncture, bipuncture, or a central venous catheter. The median [Q1–Q3] ultrafiltration volume was 550 [375–1,200] mL and the median [Q1–Q3] blood flow rate was 142 [110–260] mL/min.

All patients had MSSA bloodstream infection. No MIC was available.

Cefazolin was administered by 30–60 min intravenous intermittent infusions with various dosing regimens. The median cefazolin doses per infusion ranged from 4.9 to 26.3 mg/kg.

All patients recovered from their MSSA infection with sterilized blood cultures.

### Population PK modeling

**Fig 1:**
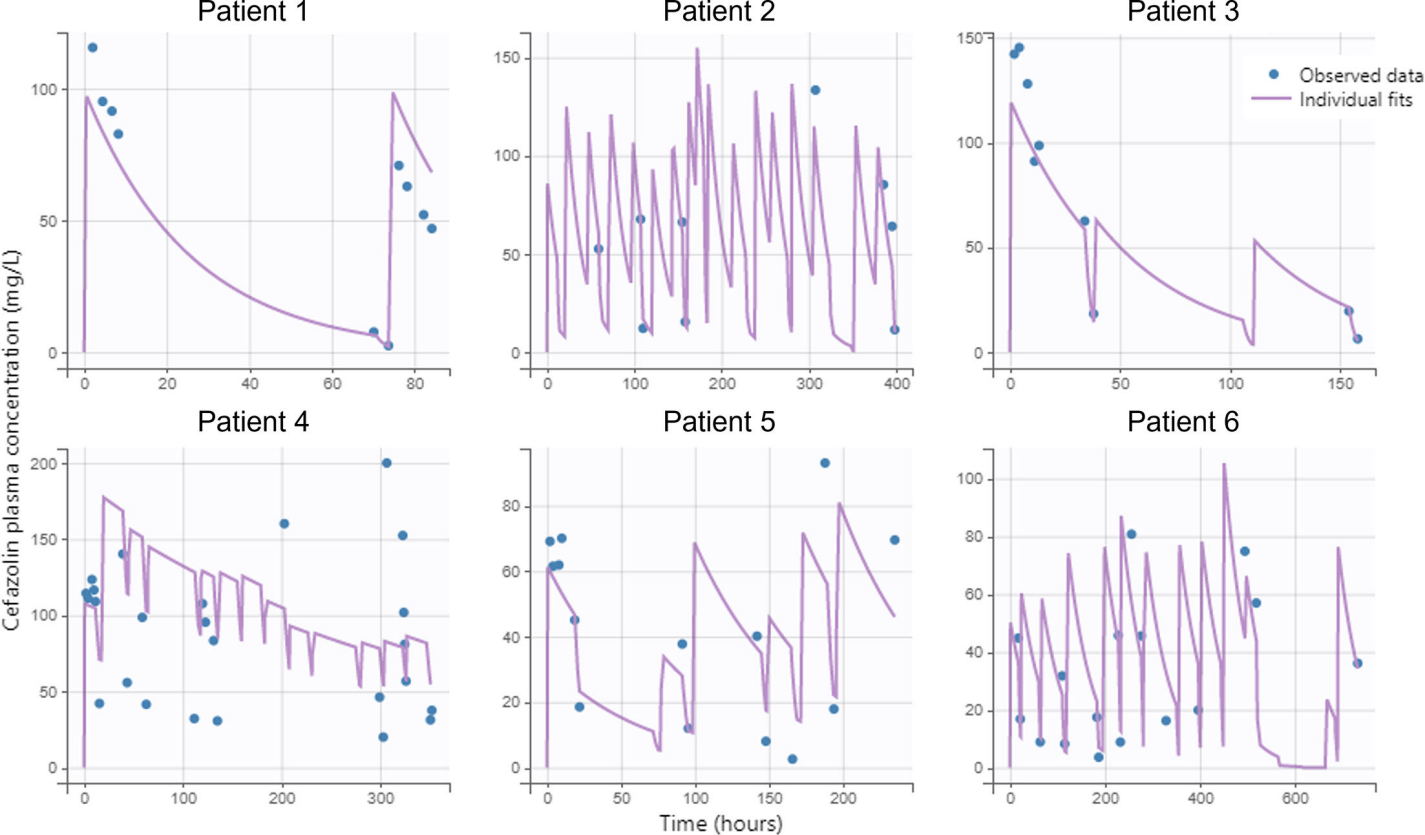
Observed data and individual fits for each subject. *Blue circles* are observations, total cefazolin plasma concentrations, in mg/L; time is in hours (h). The *continuous purple line* stands for the curve from simulations using the design and the covariates of each subject: the predicted profile given by the estimated individual model.

**Fig 2:**
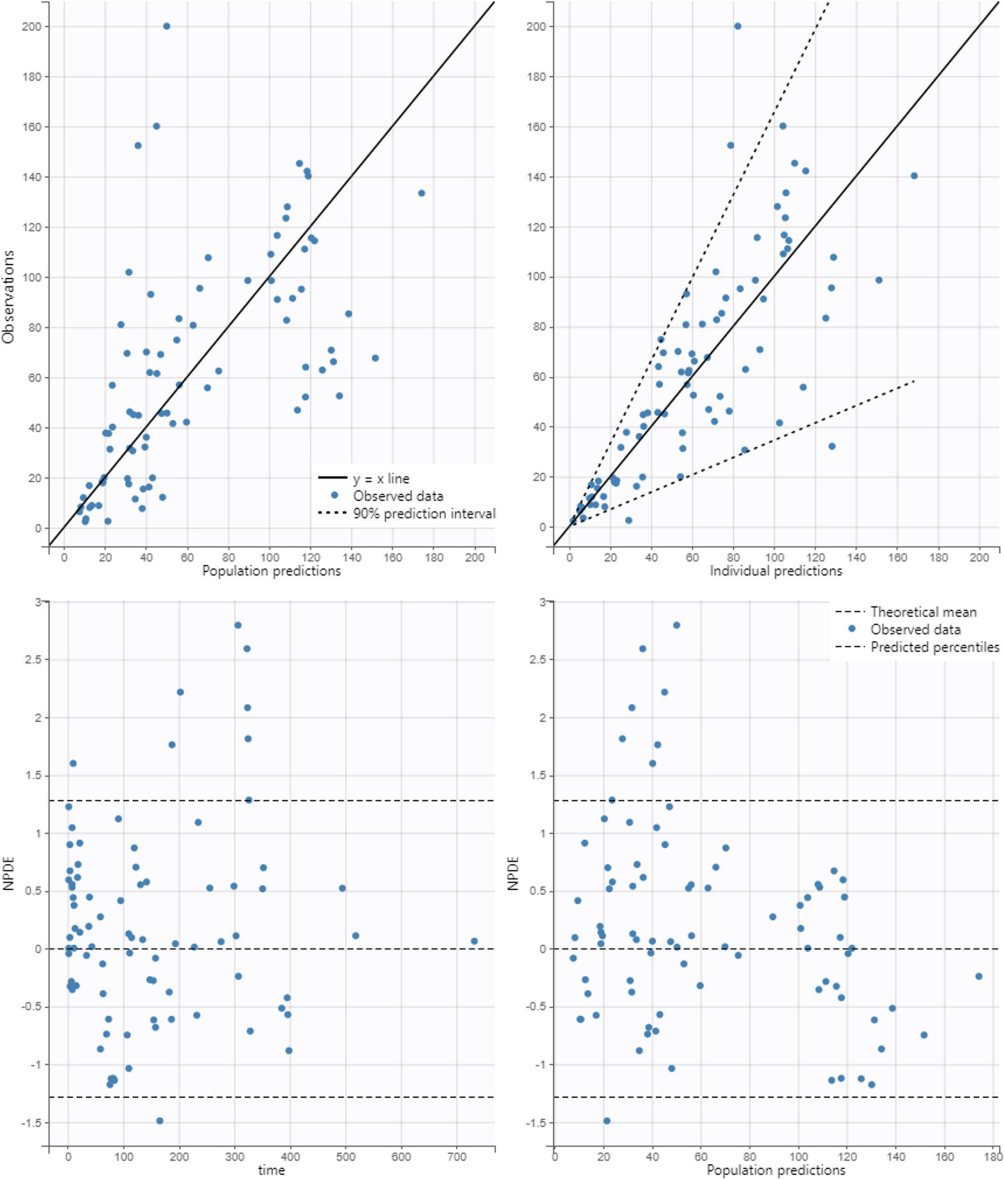
Goodness-of-fit plots for the final cefazolin population model. The *continuous black line* stands for the *y* = *x* line (top) and the *dashed black lines* stand for the 90% prediction interval of cefazolin total plasma concentrations in mg/L (top), the *y* = 0 line and predicted percentiles of normalized prediction distribution error (NPDE) (bottom). *Observations* stands for cefazolin concentrations in mg/L*. Population predictions* and *individual predictions* stand for population and individual predicted cefazolin concentrations in mg/L.

**Fig 3:**
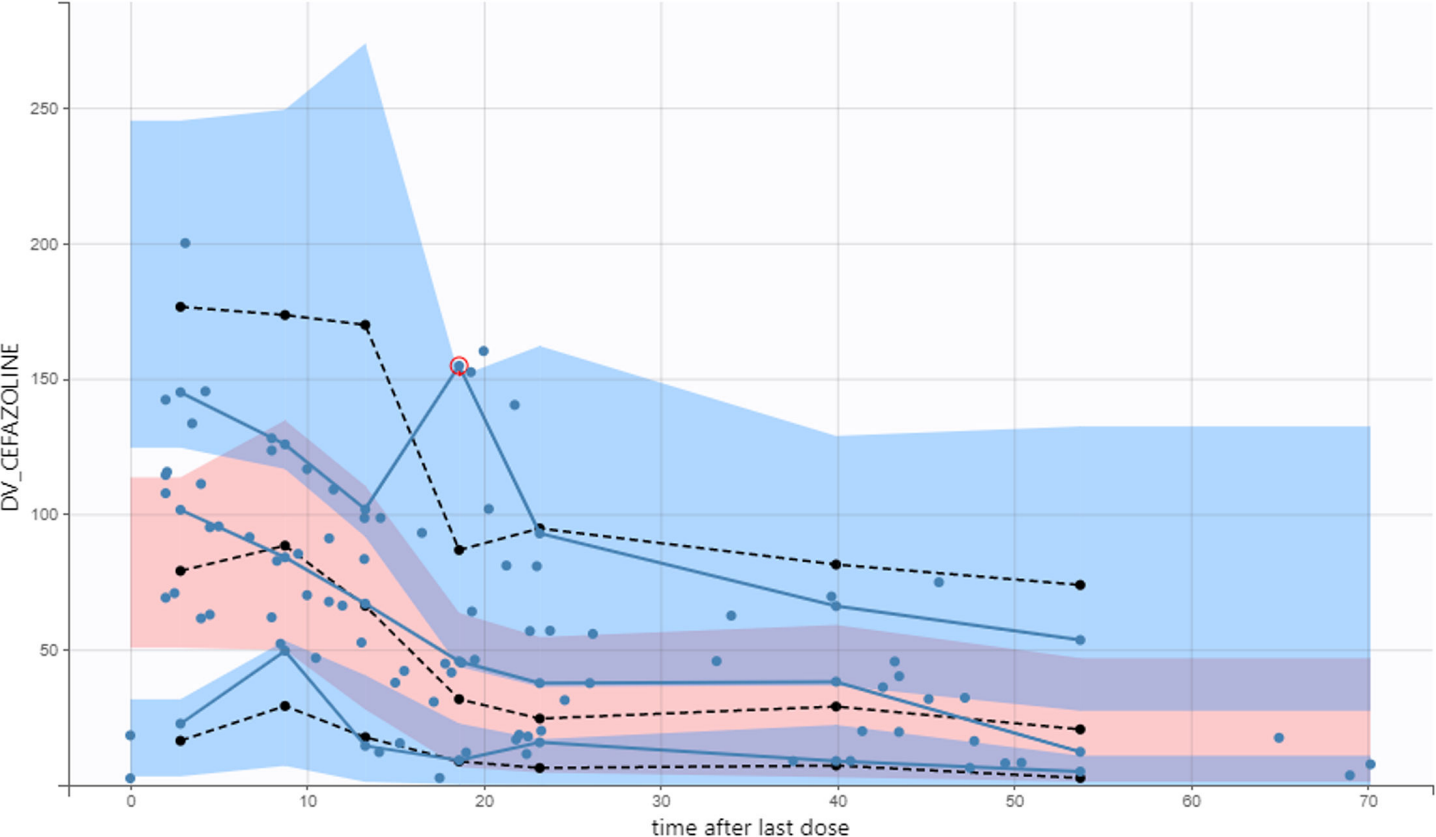
Prediction-corrected visual predictive check, total cefazolin plasma concentration versus time after last dose. *Solid and dashed lines* depict the 5th, 50th, and 95th percentiles of observed and predicted concentrations, respectively. The *blue* and *light-red areas* represent the corresponding 90th confidence intervals of the predictions. *Blue circles* are observations, total cefazolin plasma concentrations, in mg/L; time is in hours (h).

A one-compartment model with first-order elimination best described the data. The BSV could be estimated for both CL and Vd. A proportional model was used to describe the residual variability. The BIC decreased by 14 units when using the weight-based allometric approach compared to the base model. The BSV decreased from 1.42 to 1.08, from 0.95 to 0.62, and from 0.51 to 0.2 for CL, CLdial, and Vd, respectively. Adding dialysis membrane surface area (DMSA) (in m²) effect on CLdial made the effect of allometrically scaled BW on CLdial non-significant, further decreased the BIC by six units and reduced the BSV on CLdial towards 0 suggesting that DMSA explained most of the interindividual variability on that parameter. Weight-based allometric approach on CLdial was not retained in the final model. No significant inter-occasion variability was observed. The final PK parameter estimates are summarized in Table 2. Population dialysis clearance was on average more than 10 times the population residual clearance. The other covariates, including serum albumin concentration and age, had no significant effect. Individual fits of the model are presented in Fig. 1. The goodness-of-fit plots are depicted in Fig. 2. Prediction-corrected visual predictive check (pc-VPC) is shown in Fig. 3.

**TABLE 2:** Estimates of the final population pharmacokinetic parameters of cefazolin, standardized for a body weight of 70 kg^*a*^

Parameter	Estimate	RSE (%)	Shrinkage (%)
Structural model			
CL_pop_ (L/h)	0.186	45.7	
β_BW/CL_ (BW/70)^β^	0.75	Fixed	
CLdial_pop_ (L/h)	1.98	3.74	
Vd_pop_ (L)	14.6	10.9	
β_BW/Vd_ (BW/70)^β^	1	Fixed	
β_DMSA/CLdial_ (DMSA/1)^β^	1.26	1.06	
Statistical model			
ωCL	1.07	33.7	8.43
ωVd	0.197	49.9	20.6
Residual variability			
*b* (proportional)	0.398	9.33	

^*a*^ CL_pop_, population residual elimination clearance (in L/h); BW, body weight (in kg); CLdial_pop_, population dialysis clearance (in L/h); Vd_pop_, volume of distribution (in L); DMSA, dialysis membrane surface area (in m^2^); β_BW/CL_, exponent quantifying the effect of body weight on residual elimination clearance; β_BW/Vd_, exponent quantifying the effect of body weight on volume of distribution; β_DMSA/CLdial_, exponent quantifying the effect of dialysis membrane surface area on dialysis clearance; ω, between-subject variability - square root of the between-subjects variance ω² - on residual elimination clearance and volume of distribution; RSE, relative standard errors.

For the *i*^th^ patient, weighting BW*_i_* normalized by 70 kg, the final equations were:


$$Vd_{i}(L)=14.6\times ({BW}_{i}/70)$$

$$CL_{i}(L/h)=0.186\times ({BW}_{i}/70{)}^{0.75}$$

$$CLdial_{i}(L/h)=1.98\times ({DMSA}_{i}/1{)}^{1.26}$$

$${CLtot}_{i}={CL}_{i}+{CLdial}_{i}$$

### Dosing regimen simulations

Using our final model, we performed Monte Carlo simulations to determine the best dosing regimen to optimize cefazolin plasma concentrations. We simulated various dosing regimens targeting both *C* = 20–80 mg/L and *C* = 40–80 mg/L, corresponding to MSSA MIC of 1 and 2 mg/L, respectively.

As routinely done in clinical practice, the DMSA was implemented as a function of body surface area (BSA) which was calculated using BW as follows:


$$DMSA\;(m^{2})=0.85\times BSA$$

with *BSA* in m²


$$BSA(m^{2})=[(4\times BW)+7]/(BW+90)$$

with *BW* in kg

CL was low but had a significant impact on cefazolin plasma concentrations.

Tables S1 and S2 summarize the impact of CL, categorized into six ranges, on simulated dosing regimens, for the 20–80 mg/L and the 40–80 mg/L cefazolin concentration targets, respectively.

**Fig 4:**
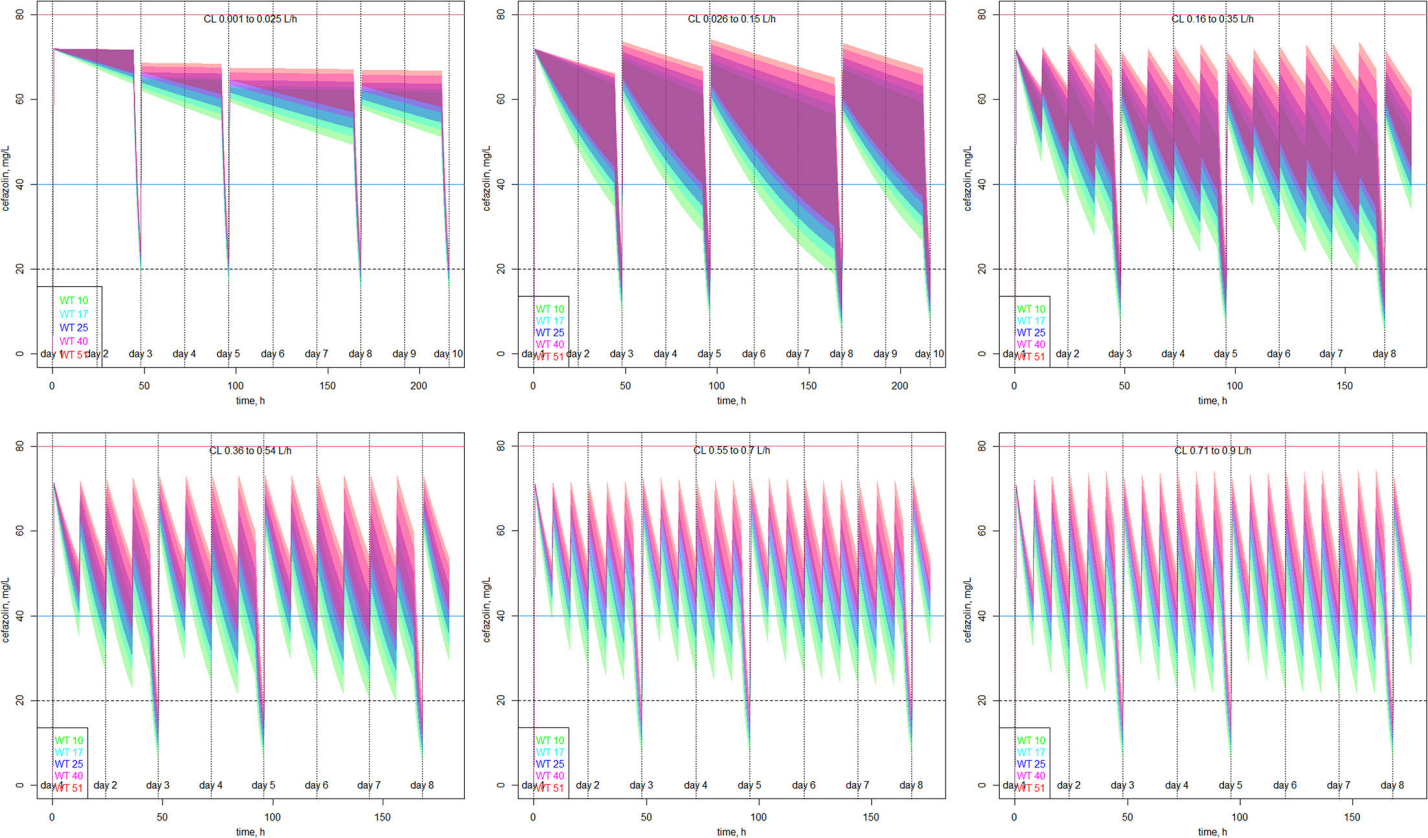
Simulations of dosing regimens based on three dialysis sessions per week (days 3, 5 and 8), for children weighing 10–51 kg and for each of the six intervals of residual elimination clearance, to optimize the time spent within the targeted concentration range 20–80 mg/L.

For the 20–80 mg/L cefazolin concentration target, the simulated daily doses over a week were 4.7, 5.1, 8.4, 12.0, 16.2, and 19.4 mg/kg/day for the [0.001–0.025], [0.026–0.15], [0.16–0.35], [0.36–0.54], [0.55–0.7], and [0.71–0.9] L/h CL ranges, respectively. The simulated mg/kg dose was the same across BWs within each CL range. Dosing regimen simulations are illustrated in Fig. 4.

For the 40–80 mg/L cefazolin concentration target, five BW ranges had to be taken into account in each CL range to improve the results. Even with different dosing regimens for each BW and CL range, the target was not optimally reached.

## DISCUSSION

To our knowledge, this is the first population PK model of cefazolin in children undergoing maintenance hemodialysis for kidney failure. This is a potentially major advance that could improve the management of children in situations that are always critical and for which clinicians were previously uncertain due to the absence of a reliable recommendation. Our model underlines a significant BSV and describes the effects of BW and of the DMSA on cefazolin PK parameters.

No recent data exists on cefazolin PK in children undergoing maintenance hemodialysis. As expected, CL was much lower than that observed in normo-renal children (25, 26, 47, 48). Interestingly, it was also similar to non-renal interdialytic cefazolin clearance observed by Schmitz et al. in pediatric and adult patients receiving cefazolin in a perioperative prophylaxis setting (48). Our interdialytic cefazolin half-life was almost twice as high as that observed in adults (49, 50) and children undergoing maintenance hemodialysis in historical papers (38, 39).

In our cohort, the cefazolin Vd was within a very large range observed in normo-renal children (26, 47, 51) and slightly higher than that observed in adults undergoing hemodialysis (21, 50). No comparative data were available for children undergoing maintenance hemodialysis.

Cefazolin CLdial was very close, and cefazolin dialysis half-life was slightly higher to those observed in adults and children undergoing maintenance hemodialysis (21, 39, 49, 50, 52). We implemented DMSA as 85% of BSA in our dosing regimen simulations as it is close to the middle of the recommended range, which is 75–100% of BSA (53–55), and as it is the recommended coefficient used to initiate dialysis in our center. In the event that a different coefficient is used for DMSA, CLdial would change proportionally and dosing regimen would have to be adjusted accordingly, particularly the post-dialysis dose.

As previously shown in prophylactic use (25, 47, 51) and in the only recent PK study on curative use of cefazolin in critically ill children (26), BW was the main covariate. Even if we were not able to estimate precisely the residual GFR of our patients, we found, as previous studies (25, 26, 47, 51), some clues of its importance in cefazolin PK. Interestingly, patient 4 had undergone a bilateral nephrectomy, which allowed us to observe and quantify a significant non-renal interdialytic clearance of cefazolin.

Although the patients’ GFR could not be precisely calculated at the time of the study, all had been on maintenance hemodialysis for several months and had their GFR estimated at various times prior to their staphylococcal infection. All were below 15 mL/min/1.73 m² (=0.9 L/h/1.73 m^2^), the usual threshold below which it is recommended to consider starting dialysis (56). Surprisingly, our data showed that CL still had a great importance even in this population of children who were all undergoing maintenance hemodialysis for kidney failure with low GFR and who were most often only categorized as “dialyzed” in dosing recommendations.

We chose to aim for the recommended target concentrations in critically ill patients because of the great vulnerability of our population due to their dependence on dialysis, the precariousness of their vascular access, and their susceptibility to severe infections (2–4, 57).

Although it has been reduced using the weight-based allometric approach, the BSV on CL remained large. As illustrated in Tables S1 and S2, the only way to achieve an acceptable proportion of cefazolin concentration-time courses within the target was to simulate different dosing regimens for different ranges of CL. For the 40–80 mg/L range, it was necessary to consider different BW ranges within each CL range, and yet the target attainment was not optimal. Making a priori dosing regimen recommendations would therefore have been too imprecise, especially since patients’ cefazolin residual elimination clearance (CL) is not available in clinical settings. Accordingly, this highlights the inappropriateness of current dosing recommendations (58) as well as the urgent need for therapeutic drug monitoring (TDM). Indeed, TDM, which consists of measuring the patients’ plasma concentration of cefazolin, is the only way to accurately determine each patient’s individual CL, as well as to take into account each patient’s specificities (27) that cannot be precisely anticipated a priori, such as protein binding change because of serum albumin concentration variation, fluctuating Vd due to kidney failure, to dialysis sessions, frequent denutrition, sarcopenia, and so on.

As expected, DMSA was found to affect CLdial in our model, as previously reported (27, 28).

Despite the strong binding of cefazolin (around 80%) to albumin and the relatively wide range of serum albumin levels in our patients, the addition of this parameter did not improve the model, nor did the use of FFM instead of total BW. This is likely due to a lack of power linked to the small number of patients in our population.

Our study has limitations. The low number of patients is one that was expected as maintenance hemodialysis is rare in children, and MSSA infections are fortunately also rare in children undergoing maintenance hemodialysis. By the way, no patient on maintenance hemodialysis has had a MSSA infection since January 2020 in our center. We minimized as much as possible this limitation by having a significant number of samples from each patient and centralized single laboratory cefazolin assay. The observations from patient 4 are not perfectly described by the model (Fig. 1). This was expected since patient 4 is an extreme case. He is the patient who had undergone a bilateral nephrectomy and was therefore anephric. His individual clearance of cefazolin was very low (0.0073 L/h), almost five times lower than the second lowest cefazolin residual clearance. This patient was very difficult to dialyze, and his hemodynamic tolerance to dialysis sessions was very poor. Another limitation is our inability to describe the association between cefazolin plasma concentration and clinical or bacteriological outcomes. This is due to the small size of our cohort and to the fact that maintenance hemodialysis patients are often treated on an outpatient basis, preventing the collection of detailed accurate clinical and bacteriological data. No MIC was available either. We minimized the impact of this unavailability by simulating dosing regimens for both EUCAST’s ECOFF for *Staphylococcus aureus* and another relevant MIC threshold value.

However, our study improved the knowledge on cefazolin PK in the specific population of children undergoing maintenance hemodialysis for kidney failure, showing a high BSV and the inappropriateness of currently used dosing regimen, highlighting the impact of BW and DMSA on PK parameters, and the unexpected importance of CL, even when low, on cefazolin plasma concentration and therefore on dosing regimens (57).

### Conclusion

To optimize cefazolin exposure in children on maintenance hemodialysis for kidney failure, dosing regimen needs to be individualized according to BW, DMSA, and CL. Due to the persistence of a large unexplained BSV on CL that substantially affects cefazolin concentrations, proposing accurate dosing regimens a priori appears difficult. Therefore, the optimal dosing scheme should be individually determined using TDM.

## SUPPLEMENTAL MATERIAL

Supplemental material aac.00451-25-s0001.docx Tables S1 and S2.

ASM does not own the copyrights to Supplemental Material that may be linked to, or accessed through, an article. The authors have granted ASM a non-exclusive, world-wide license to publish the Supplemental Material files. Please contact the corresponding author directly for reuse.
