# Supplementary material for: Cefazolin population pharmacokinetics in children undergoing maintenance hemodialysis for kidney failure
Source: Antimicrob Agents Chemother. 2025 Oct 2;69(11):e00451-25. doi: 10.1128/aac.00451-25 (PMC12587577; doi:10.1128/aac.00451-25)
Supplement: Supplemental material — Tables S1 and S2. [file aac.00451-25-s0001.docx]

**Table S1** Impact of cefazolin residual elimination clearance on simulated dosing regimen based on three dialysis sessions per week (Monday, Wednesday, Friday) for children weighing 10 to 51 kilograms, and proportion of time spent within the targeted concentration range 20-80 mg/L (pharmacological target for MICs ≤ 1mg/L) over a whole week.

| **CL, L/h** | **Administration**  **frequency** | **Dosing regimen for each day, mg/kg** | | | | | | | | **Time within the target, %** |
| --- | --- | --- | --- | --- | --- | --- | --- | --- | --- | --- |
|  |  | **Monday (D)** | **Tuesday** | **Wednesday (D)** | **Thursday** | **Friday (D)** | **Saturday** | **Sunday** | **Monday (D)** |  |
| **0.001-0.025** | /48h* | 15.0 | 0 | 9.0 | 0 | 9.0 | 0 | 0 | 9.0 | 99.5 |
| **0.026-0.15** | /48h* | 15.0 | 0 | 10.5 | 0 | 10.5 | 0 | 0 | 10.5 | 97.1 |
| **0.16-0.35** | /12h | 15.0 / 2.1 | 2.1 / 2.1 | 10.2 / 2.1 | 2.1 / 2.1 | 10.2 / 2.1 | 2.1 / 2.1 | 2.1 / 2.1 | 10.2 / 2.1 | 97.2 |
| **0.36-0.54** | /12h | 15.0 / 4.2 | 4.2 / 4.2 | 11.4 / 4.2 | 4.2 / 4.2 | 11.4 / 4.2 | 4.2 / 4.2 | 4.2 / 4.2 | 11.4 / 4.2 | 96.8 |
| **0.55-0.7** | /8h | 15.0 / 4.2 / 4.2 | 4.2 / 4.2 / 4.2 | 11.4 / 4.2 / 4.2 | 4.2 / 4.2 / 4.2 | 11.4 / 4.2 / 4.2 | 4.2 / 4.2 / 4.2 | 4.2 / 4.2 / 4.2 | 11.4 / 4.2 / 4.2 | 97.3 |
| **0.71-0.9** | /8h | 15.0 / 5.4 / 5.4 | 5.4 / 5.4 / 5.4 | 11.7 / 5.4 / 5.4 | 5.4 / 5.4 / 5.4 | 11.7 / 5.4 / 5.4 | 5.4 / 5.4 / 5.4 | 5.4 / 5.4 / 5.4 | 11.7 / 5.4 / 5.4 | 97.0 |

MICs, minimum inhibitory concentrations; CL, cefazolin residual elimination clearance; (D), dialysis session; *except on Sundays, resulting in a 72-hours interval between Fridays and Mondays dialyses.

**Table S2** Impact of body weight and cefazolin residual elimination clearance on simulated dosing regimen based on three dialysis sessions per week (Monday, Wednesday, Friday) for children weighing 10 to 51 kg, and proportion of time spent within the targeted concentration range 40-80 mg/L (pharmacological target for MICs ≤ 2mg/L) over a whole week.

| **BW, kg** | **CL, L/h** | **Admin. frequency** | **Dosing regimen for each day, mg/kg** | | | | | | | | **Time**  **within the target, %** |
| --- | --- | --- | --- | --- | --- | --- | --- | --- | --- | --- | --- |
|  |  |  | **Monday (D)** | **Tuesday** | **Wednesday (D)** | **Thursday** | **Friday (D)** | **Saturday** | **Sunday** | **Monday (D)** |  |
| **10-12** | 0.001-0.025 | /48h* | 15.0 | 0 | 9.0 | 0 | 9.0 | 0 | 0 | 9.0 | 94.6 |
|  | 0.026-0.15 | /12h | 15.0 / 0.4 | 0.4 / 0.4 | 11.0 / 0.4 | 0.4 / 0.4 | 11.0 / 0.4 | 0.4 / 0.4 | 0.4 / 0.4 | 11.0 / 0.4 | 81.3 |
|  | 0.16-0.35 | /8h | 15.0 / 1.9 / 1.9 | 1.9 / 1.9 / 1.9 | 11.4 / 1.9 / 1.9 | 1.9 / 1.9 / 1.9 | 11.4 / 1.9 / 1.9 | 1.9 / 1.9 / 1.9 | 1.9 / 1.9 / 1.9 | 11.4 / 1.9 / 1.9 | 76.3 |
|  | 0.36-0.54 | /6h | 15.0 / 3.3 / 3.3 / 3.3 | 3.3 / 3.3 / 3.3 / 3.3 | 11.7 / 3.3 / 3.3 / 3.3 | 3.3 / 3.3 / 3.3 / 3.3 | 11.7 / 3.3 / 3.3 / 3.3 | 3.3 / 3.3 / 3.3 / 3.3 | 3.3 / 3.3 / 3.3 / 3.3 | 11.7 / 3.3 / 3.3 / 3.3 | 93.0 |
|  | 0.55-0.7 | /6h | 15.0 / 4.8 / 4.8 / 4.8 | 4.8 / 4.8 / 4.8 / 4.8 | 12.0 / 4.8 / 4.8 / 4.8 | 4.8 / 4.8 / 4.8 / 4.8 | 12.0 / 4.8 / 4.8 / 4.8 | 4.8 / 4.8 / 4.8 / 4.8 | 4.8 / 4.8 / 4.8 / 4.8 | 12.0 / 4.8 / 4.8 / 4.8 | 94.4 |
|  | 0.71-0.9 | /4h | 15.0 / 4.2 / 4.2 / 4.2 / 4.2 / 4.2 | 4.2 / 4.2 / 4.2 / 4.2 / 4.2 / 4.2 | 12.0 / 4.2 / 4.2 / 4.2 / 4.2 / 4.2 | 4.2 / 4.2 / 4.2 / 4.2 / 4.2 / 4.2 | 12.0 / 4.2 / 4.2 / 4.2 / 4.2 / 4.2 | 4.2 / 4.2 / 4.2 / 4.2 / 4.2 / 4.2 | 4.2 / 4.2 / 4.2 / 4.2 / 4.2 / 4.2 | 12.0 / 4.2 / 4.2 / 4.2 / 4.2 / 4.2 | 95.1 |
| **13-17** | 0.001-0.025 | /48h* | 15.0 | 0 | 9.0 | 0 | 9.0 | 0 | 0 | 9.0 | 94.7 |
|  | 0.026-0.15 | /12h | 15.0 / 0.4 | 0.4 / 0.4 | 10.8 / 0.4 | 0.4 / 0.4 | 10.8 / 0.4 | 0.4 / 0.4 | 0.4 / 0.4 | 10.8 / 0.4 | 83.9 |
|  | 0.16-0.35 | /8h | 15.0 / 1.8 / 1.8 | 1.8 / 1.8 / 1.8 | 11.1 / 1.8 / 1.8 | 1.8 / 1.8 / 1.8 | 11.1 / 1.8 / 1.8 | 1.8 / 1.8 / 1.8 | 1.8 / 1.8 / 1.8 | 11.1 / 1.8 / 1.8 | 79.0 |
|  | 0.36-0.54 | /6h | 15.0 / 3.0 / 3.0 / 3.0 | 3.0 / 3.0 / 3.0 / 3.0 | 11.7 / 3.0 / 3.0 / 3.0 | 3.0 / 3.0 / 3.0 / 3.0 | 11.7 / 3.0 / 3.0 / 3.0 | 3.0 / 3.0 / 3.0 / 3.0 | 3.0 / 3.0 / 3.0 / 3.0 | 11.7 / 3.0 / 3.0 / 3.0 | 92.8 |
|  | 0.55-0.7 | /6h | 15.0 / 4.5 / 4.5 / 4.5 | 4.5 / 4.5 / 4.5 / 4.5 | 12.3 / 4.5 / 4.5 / 4.5 | 4.5 / 4.5 / 4.5 / 4.5 | 12.3 / 4.5 / 4.5 / 4.5 | 4.5 / 4.5 / 4.5 / 4.5 | 4.5 / 4.5 / 4.5 / 4.5 | 12.3 / 4.5 / 4.5 / 4.5 | 94.7 |
|  | 0.71-0.9 | /4h | 15.0 / 3.9 / 3.9 / 3.9 / 3.9 / 3.9 | 3.9 / 3.9 / 3.9 / 3.9 / 3.9 / 3.9 | 11.7 / 3.9 / 3.9 / 3.9 / 3.9 / 3.9 | 3.9 / 3.9 / 3.9 / 3.9 / 3.9 / 3.9 | 11.7 / 3.9 / 3.9 / 3.9 / 3.9 / 3.9 | 3.9 / 3.9 / 3.9 / 3.9 / 3.9 / 3.9 | 3.9 / 3.9 / 3.9 / 3.9 / 3.9 / 3.9 | 11.7 / 3.9 / 3.9 / 3.9 / 3.9 / 3.9 | 95.2 |
| **18-24** | 0.001-0.025 | /48h* | 15.0 | 0 | 9.0 | 0 | 9.0 | 0 | 0 | 9.0 | 94.9 |
|  | 0.026-0.15 | /12h | 15.0 / 0.4 | 0.4 / 0.4 | 10.5 / 0.4 | 0.4 / 0.4 | 10.5 / 0.4 | 0.4 / 0.4 | 0.4 / 0.4 | 10.5 / 0.4 | 86.4 |
|  | 0.16-0.35 | /8h | 15.0 / 1.6 / 1.6 | 1.6 / 1.6 / 1.6 | 11.2 / 1.6 / 1.6 | 1.6 / 1.6 / 1.6 | 11.2 / 1.6 / 1.6 | 1.6 / 1.6 / 1.6 | 1.6 / 1.6 / 1.6 | 11.2 / 1.6 / 1.6 | 80.5 |
|  | 0.36-0.54 | /6h | 15.0 / 2.8 / 2.8 / 2.8 | 2.8 / 2.8 / 2.8 / 2.8 | 11.4 / 2.8 / 2.8 / 2.8 | 2.8 / 2.8 / 2.8 / 2.8 | 11.4 / 2.8 / 2.8 / 2.8 | 2.8 / 2.8 / 2.8 / 2.8 | 2.8 / 2.8 / 2.8 / 2.8 | 11.4 / 2.8 / 2.8 / 2.8 | 93.8 |
|  | 0.55-0.7 | /6h | 15.0 / 4.0 / 4.0 / 4.0 | 4.0 / 4.0 / 4.0 / 4.0 | 11.7 / 4.0 / 4.0 / 4.0 | 4.0 / 4.0 / 4.0 / 4.0 | 11.7 / 4.0 / 4.0 / 4.0 | 4.0 / 4.0 / 4.0 / 4.0 | 4.0 / 4.0 / 4.0 / 4.0 | 11.7 / 4.0 / 4.0 / 4.0 | 94.5 |
|  | 0.71-0.9 | /4h | 15.0 / 3.6 / 3.6 / 3.6 / 3.6 / 3.6 | 3.6 / 3.6 / 3.6 / 3.6 / 3.6 / 3.6 | 11.7 / 3.6 / 3.6 / 3.6 / 3.6 / 3.6 | 3.6 / 3.6 / 3.6 / 3.6 / 3.6 / 3.6 | 11.7 / 3.6 / 3.6 / 3.6 / 3.6 / 3.6 | 3.6 / 3.6 / 3.6 / 3.6 / 3.6 / 3.6 | 3.6 / 3.6 / 3.6 / 3.6 / 3.6 / 3.6 | 11.7 / 3.6 / 3.6 / 3.6 / 3.6 / 3.6 | 95.2 |
| **25-39** | 0.001-0.025 | /48h* | 15.0 | 0 | 9.0 | 0 | 9.0 | 0 | 0 | 9.0 | 95.1 |
|  | 0.026-0.15 | /12h | 15.0 / 0.3 | 0.3 / 0.3 | 10.5 / 0.3 | 0.3 / 0.3 | 10.5 / 0.3 | 0.3 / 0.3 | 0.3 / 0.3 | 10.5 / 0.3 | 87.7 |
|  | 0.16-0.35 | /8h | 15.0 / 1.5 / 1.5 | 1.5 / 1.5 / 1.5 | 10.5 / 1.5 / 1.5 | 1.5 / 1.5 / 1.5 | 10.5 / 1.5 / 1.5 | 1.5 / 1.5 / 1.5 | 1.5 / 1.5 / 1.5 | 10.5 / 1.5 / 1.5 | 83.4 |
|  | 0.36-0.54 | /6h | 15.0 / 2.5 / 2.5 / 2.5 | 2.5 / 2.5 / 2.5 / 2.5 | 11.2 / 2.5 / 2.5 / 2.5 | 2.5 / 2.5 / 2.5 / 2.5 | 11.2 / 2.5 / 2.5 / 2.5 | 2.5 / 2.5 / 2.5 / 2.5 | 2.5 / 2.5 / 2.5 / 2.5 | 11.2 / 2.5 / 2.5 / 2.5 | 93.7 |
|  | 0.55-0.7 | /6h | 15.0 / 3.6 / 3.6 / 3.6 | 3.6 / 3.6 / 3.6 / 3.6 | 11.2 / 3.6 / 3.6 / 3.6 | 3.6 / 3.6 / 3.6 / 3.6 | 11.2 / 3.6 / 3.6 / 3.6 | 3.6 / 3.6 / 3.6 / 3.6 | 3.6 / 3.6 / 3.6 / 3.6 | 11.2 / 3.6 / 3.6 / 3.6 | 94.4 |
|  | 0.71-0.9 | /4h | 15.0 / 3.2 / 3.2 / 3.2 / 3.2 / 3.2 | 3.2 / 3.2 / 3.2 / 3.2 / 3.2 / 3.2 | 11.2 / 3.2 / 3.2 / 3.2 / 3.2 / 3.2 | 3.2 / 3.2 / 3.2 / 3.2 / 3.2 / 3.2 | 11.2 / 3.2 / 3.2 / 3.2 / 3.2 / 3.2 | 3.2 / 3.2 / 3.2 / 3.2 / 3.2 / 3.2 | 3.2 / 3.2 / 3.2 / 3.2 / 3.2 / 3.2 | 11.2 / 3.2 / 3.2 / 3.2 / 3.2 / 3.2 | 95.2 |
| **40-51** | 0.001-0.025 | /48h* | 15.0 | 0 | 9.0 | 0 | 9.0 | 0 | 0 | 9.0 | 95.5 |
|  | 0.026-0.15 | /12h | 15.0 / 0.3 | 0.3 / 0.3 | 10.0 / 0.3 | 0.3 / 0.3 | 10.0 / 0.3 | 0.3 / 0.3 | 0.3 / 0.3 | 10.0 / 0.3 | 89.2 |
|  | 0.16-0.35 | /8h | 15.0 / 1.4 / 1.4 | 1.4 / 1.4 / 1.4 | 10.2 / 1.4 / 1.4 | 1.4 / 1.4 / 1.4 | 10.2 / 1.4 / 1.4 | 1.4 / 1.4 / 1.4 | 1.4 / 1.4 / 1.4 | 10.2 / 1.4 / 1.4 | 86.4 |
|  | 0.36-0.54 | /6h | 15.0 / 2.4 / 2.4 / 2.4 | 2.4 / 2.4 / 2.4 / 2.4 | 10.3 / 2.4 / 2.4 / 2.4 | 2.4 / 2.4 / 2.4 / 2.4 | 10.3 / 2.4 / 2.4 / 2.4 | 2.4 / 2.4 / 2.4 / 2.4 | 2.4 / 2.4 / 2.4 / 2.4 | 10.3 / 2.4 / 2.4 / 2.4 | 94.9 |
|  | 0.55-0.7 | /6h | 15.0 / 3.4 / 3.4 / 3.4 | 3.4 / 3.4 / 3.4 / 3.4 | 11.1 / 3.4 / 3.4 / 3.4 | 3.4 / 3.4 / 3.4 / 3.4 | 11.1 / 3.4 / 3.4 / 3.4 | 3.4 / 3.4 / 3.4 / 3.4 | 3.4 / 3.4 / 3.4 / 3.4 | 11.1 / 3.4 / 3.4 / 3.4 | 95.0 |
|  | 0.71-0.9 | /4h | 15.0 / 3.0 / 3.0 / 3.0 / 3.0 / 3.0 | 3.0 / 3.0 / 3.0 / 3.0 / 3.0 / 3.0 | 10.9 / 3.0 / 3.0 / 3.0 / 3.0 / 3.0 | 3.0 / 3.0 / 3.0 / 3.0 / 3.0 / 3.0 | 10.9 / 3.0 / 3.0 / 3.0 / 3.0 / 3.0 | 3.0 / 3.0 / 3.0 / 3.0 / 3.0 / 3.0 | 3.0 / 3.0 / 3.0 / 3.0 / 3.0 / 3.0 | 10.9 / 3.0 / 3.0 / 3.0 / 3.0 / 3.0 | 95.4 |

Admin, administration; MICs, minimum inhibitory concentrations; BW, body weight; CL, cefazolin residual elimination clearance; (D), dialysis session; *except on Sundays, resulting in a 72-hours interval between Fridays and Mondays dialyses.
